# Classification of Diabetic Foot Ulcers from Images Using Machine Learning Approach

**DOI:** 10.3390/diagnostics14161807

**Published:** 2024-08-19

**Authors:** Nouf Almufadi, Haifa F. Alhasson

**Affiliations:** Department of Information Technology, College of Computer, Qassim University, Buraydah 51452, Saudi Arabia

**Keywords:** diabetics, diabetic foot ulcers, convolutional neural networks, deep learning, diagnosis, transfer learning, image classifier

## Abstract

Diabetic foot ulcers (DFUs) represent a significant and serious challenge associated with diabetes. It is estimated that approximately one third of individuals with diabetes will develop DFUs at some point in their lives. This common complication can lead to serious health issues if not properly managed. The early diagnosis and treatment of DFUs are crucial to prevent severe complications, including lower limb amputation. DFUs can be categorized into two states: ischemia and infection. Accurate classification is required to avoid misdiagnosis due to the similarities between these two states. Several convolutional neural network (CNN) models have been used and pre-trained through transfer learning. These models underwent evaluation with hyperparameter tuning for the binary classification of different states of DFUs, such as ischemia and infection. This study aimed to develop an effective classification system for DFUs using CNN models and machine learning classifiers utilizing various CNN models, such as EfficientNetB0, DenseNet121, ResNet101, VGG16, InceptionV3, MobileNetV2, and InceptionResNetV2, due to their excellent performance in diverse computer vision tasks. Additionally, the head model functions as the ultimate component for making decisions in the model, utilizing data collected from preceding layers to make precise predictions or classifications. The results of the CNN models with the suggested head model have been used in different machine learning classifiers to determine which ones are most effective for enhancing the performance of each CNN model. The most optimal outcome in categorizing ischemia is a 97% accuracy rate. This was accomplished by integrating the suggested head model with the EfficientNetB0 model and inputting the outcomes into the logistic regression classifier. The EfficientNetB0 model, with the proposed modifications and by feeding the outcomes to the AdaBoost classifier, attains an accuracy of 93% in classifying infections.

## 1. Introduction

Diabetes is a chronic condition caused by high blood sugar levels. There are two main types: one due to insufficient insulin production and the other from ineffective insulin use in the body [1]. Diabetes may lead to severe health issues that can be life-threatening, including the formation of Diabetic Foot Ulcers (DFUs) [2]. DFUs present a significant complication of diabetes, associated with peripheral vascular disease and nervous system dysfunction. The formation of DFUs results from reduced blood flow and nerve damage, which can lead to tissue damage and poor wound healing [3].

The lifetime risk of an individual with diabetes developing a DFU is approximately 34%, indicating that roughly one out of every three diabetes patients is likely to experience this complication [4]. DFUs have a high recurrence rate, with 40% after the first year of occurrence and approximately 60% within three years [4]. The mismanagement and lack of acknowledgment of diabetic foot sores can result in the need for lower limb amputation. More than one million patients with diabetes in the United States undergo amputation as a result of diabetes each year [5].

The continuous assessment of patients with DFUs is crucial for healthcare professionals to monitor healing progress and determine the most suitable medication to prevent complications [6,7,8]. DFUs can be attributed to two primary factors: ischemia, resulting from limited blood flow, and infection, stemming from bacterial invasion in the affected wound sites. Limb loss within three years is a risk for 40% of patients with ischemia, mainly due to the potential for infection [9]. Approximately 56% of DFU cases result in infection, with 20% of these infections ultimately leading to limb amputation [10,11,12].

DFUs have a negative impact on a patient’s quality of life and lead to an increased economic burden. Improved classification methods and the early identification of DFUs play crucial roles in providing timely intervention, accurate diagnosis, and efficient treatment to minimize the associated consequences. Conventional approaches for assessing DFUs, such as blood tests, physical examinations, and Doppler studies of leg blood vessels, are both expensive and time-consuming.

Automated telemedicine systems can use Machine Learning (ML) and Deep Learning (DL) techniques to assess ischemia and infection in DFUs based on the visual characteristics of images. Ischemia is indicated by tissue death leading to black gangrenous toes, while indicators of infection include purulent coloration and redness in and around the ulcer [13]. Spotting infections and ischemia is essential for evaluating the healing process and minimizing the chance of amputation.

The main contributions of this study are as follows:

- Explored and evaluated different ML models, and fed their results to modified versions of different CNNs pre-trained on the DFU-Part (B) dataset to perform the binary classification of infection and ischemia.
- Investigated the impact of feeding the results of the pre-trained models and the proposed model to different ML classifiers on enhancing the results of the models.

## 2. Related Work

The number of studies that use ML and DL algorithms to detect or classify DFU images has increased in recent years due to the importance of developing more efficient DFU assessment automatic intelligent telemedicine systems to assist in diagnosing DFUs correctly and rapidly, rather than using traditional methods, which are costly and time-consuming.

The authors of [14] presented novel DL methods for the real-time detection of DFU. The method incorporates several stages, including using a CNN as a feature extractor, the generation of proposals and refinement, and finally, a region of interest (ROI) classifier and bounding box regressor. Moreover, Cruz et al. [15] aimed to classify patterns in thermal images of patients with diabetes mellitus into five distinct levels. The study sought to evaluate the effectiveness of conventional classifiers, such as artificial neural networks (ANN) and support vector machines (SVM), as well as more contemporary and relevant classifiers, such as convolutional neural networks (CNNs) for pattern classification in thermal images. The researchers in [16] used four different models to detect DFU, including single-shot detectors MobileNet (SSD-MobileNet), SSD-InceptionV2, Faster R-CNN with InceptionV2, and R-FCN with ResNet 101. However, this study focused on classifying DFU images for the binary classification of infection and ischemia. Therefore, this section includes an overview of previous studies that focused on classifying DFU images into binary classifications of infection and ischemia using ML and DL algorithms.

An ensemble approach based on CNN was utilized in [17] to integrate different CNN pre-trained models and then feed the model results into the SVM algorithm to produce predictions. The CNN pre-trained models used were the Inception-V3 model, the InceptionResNetV2 model, and the ResNet50 model. Furthermore, the researchers proposed a new data-augmentation technique to find the image ROI from foot images and proposed a new feature descriptor that can extract the color ROIs from DFU images. The proposed ensemble CNN algorithms achieved an accuracy rate of 90% in ischemia classification and 73% in infection classification.

In contrast, a 16-layer CNN model was proposed in [18]. The deep features extracted from the proposed CNN model are supplied to different classifiers, which are Naive Bayes (NB), Softmax, Decision Tree (DT), K-nearest Neighbor (KNN), and Ensemble. The researchers found that the DT and Softmax classifiers achieved the highest results in classifying DFU images for both ischemia and infection classifications across all performance metrics. For infection classification, the DT achieved promising results with 0.996 accuracy, and Softmax achieved 0.943 accuracy. For ischemia classification, Softmax achieved 0.976 accuracy, and DT achieved 0.948 accuracy.

Al-Garaawi et al. [19] presented a method based on a CNN that feeds the CNN model texture information from DFU RGB images. The method includes two stages: using a technique named mapped binary patterns to extract texture information from the RGB image, and then using the obtained mapped image containing the texture information as input for the CNN model to recognize DFUs. The proposed method achieved an accuracy rate of 99% in recognizing ischemia and 74% in recognizing infection.

The authors of [20] proposed a system that includes several stages, including a feature extraction stage, followed by a feature fusion stage, and finally a DFU classification stage. For the feature extraction stage, different types of texture features are extracted using the GoogLeNet CNN model, including the histogram of oriented gradients (HOG), Gabor, and deep features. The feature fusion stage combines the extracted feature vectors into a single feature vector. Finally, the classification stage uses the random forest algorithm on the fusion vectors to perform DFU classification. The proposed system achieved a 92% accuracy rate in ischemia classification and 73% in infection classification.

Additionally, the researchers in [21] proposed class knowledge banks (CKBs) to extract class knowledge from the data and then store it in the CKBs to use for prediction with the input images and the trained parameters in the networks. The researchers experimented with different models with the proposed method, and the highest achieved accuracy was 78% for infection recognition, while for ischemia recognition, the highest achieved accuracy was 90.90%.

Moreover, a new CNN-based approach (ResKNet) is proposed in [22] for ischemia and infection classification. The approach includes a sequence of different layers, which are experimented with different networks, starting from Res4Net to Res10Net. The Res4Net achieved the highest result with 97.8% accuracy for ischemia recognition. For infection recognition, the highest result was achieved by the Res7Net network with 80% accuracy. Table 1 summarizes the studies that implemented binary classification for infection and ischemia.

## 3. Methods

The proposed approach was implemented using Python 3.10 in a cloud computing environment, specifically the Google Colab Pro version with a T4 GPU accelerator. The TensorFlow platform was used along with the Keras framework to implement this work, utilizing various libraries such as Sklearn, Pandas, Numpy, glob, and Matplotlib.

### 3.1. Proposed Framework

**Figure 1:**
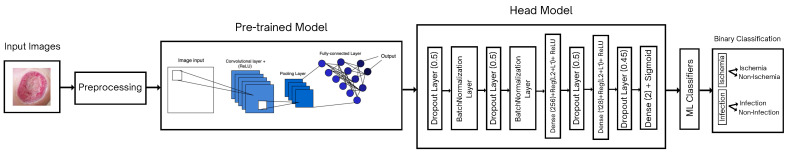
Framework of the proposed approach for infection classification and ischemia classification.

Figure 1 illustrates the proposed approach. We combined the pre-trained CNN models with the proposed head model and fed the results to various ML classifiers to find the most effective classifier to enhance the overall results. The proposed approach is used for both ischemia classification and infection classification.

### 3.2. Dataset

The DFU-Part (B) dataset [17] was used in this study. This dataset was used for performing the binary classification of ischemia and the binary classification of infection from DFU images. The dataset contains 1249 images of ischemia and 210 images of non-ischemia. Additionally, there are 628 images of infection and 831 images of non-infection. The authors of the DFU-Part (B) dataset proposed a new data augmentation technique called natural data augmentation to make the dataset balanced. The natural data augmentation is designed to identify the region of interest in DFU images. After the natural augmentation was conducted, the authors of the chosen dataset created a total of 9870 augmented images with an equal distribution of 4935 images for ischemia and 4935 images for non-ischemia. In terms of infection, they generated 4890 augmented images with an equal class distribution comprising 2945 infection images and 2945 non-infection images.

### 3.3. Pre-Processing

Due to the better performance of CNN models with larger datasets [23], we applied data augmentation to the dataset, which plays a significant role in ensuring accurate and dependable outcomes by increasing the number of images in the dataset. Furthermore, data augmentation assists in avoiding the overfitting problem [23]. The data augmentation techniques that were applied include rotation, horizontal flip, and vertical flip. Other augmentation techniques, such as crop, translation, and random scale, were avoided due to the risk of missing the region of interest in the DFU images. Table 2 illustrates the number of images in the selected dataset before and after applying the natural data augmentation and our data augmentation for both ischemia classification and infection classification using the parameter values that have been used on the dataset shown in Table 3.

The sizes of the images in the dataset varied between 1600×200 and 3648×2736 pixels. Therefore, the images were resized to 224×224 pixels to decrease the training time. The number of ischemia images in the ischemia dataset was increased from 9870 to 12,124 (ischemia [6062], non-ischemia [6062]), and that of infection images in the infection dataset was increased from 4890 to 8424 (infection [4212], non-infection [4212]). The ischemia dataset and the infection dataset were split into 70% for training, 20% for validation, and 10% for testing. The ratio of splitting the dataset into training, testing, and validation was selected after trying different ratios with the aim of picking the split ratio that provided the best results. After comparing different ratios, the ratio of 70% for training, 20% for validation, and 10% for testing was chosen as it showed the best results compared to other split ratios.

**Figure 2:**
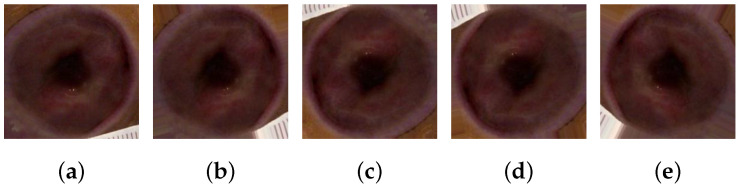
A sample of ischemia images from DFU-Part (B) dataset: (**a**) before augmentation and (**b**–**e**) newly generated ischemia images after augmentation.

**Figure 3:**
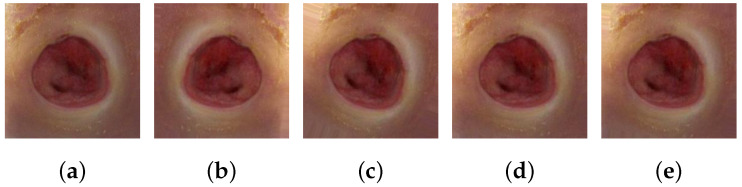
A sample of infection images from DFU-Part (B) dataset: (**a**) before augmentation, and (**b**–**e**) newly generated infection images after augmentation.

Figure 2 shows an example of an ischemia image before and after augmentation; Figure 2a is an ischemia image before augmentation, and Figure 2b–e are the newly generated ischemia images after augmentation. Moreover, Figure 3 shows an example of an infection image before and after data augmentation. Figure 3a shows an infection image before augmentation, and Figure 3b–e are the newly generated infection images after augmentation.

### 3.4. Competing Transfer Learning Models

Various pre-trained CNN models were utilized to examine and assess their effectiveness in classifying infection and ischemia images. This phase involved evaluating the performances of different CNN models independently before adding the proposed head model and feeding the models’ results to ML classifiers. The models used in this study included EfficientNetB0, DenseNet121, ResNet101, VGG16, InceptionV3, MobileNetV2, and InceptionResNetV2. The CNN models were pre-trained using the TL technique. The TL technique allows the CNN models to learn rich and discriminative features and save time and computational resources by gaining expertise from a large-scale domain and then applying it to a specific domain. The transfer learning (TL) technique has been applied to the various CNN models using the ImageNet dataset. The top layers of the pre-trained CNN model extract basic features, such as colors and edges, whereas the bottom layers capture more advanced features, such as objects and contours. In this way, we utilized the ability of the pre-trained CNN models to extract significant features and enhance classification accuracy by applying the knowledge gained from a pre-trained CNN model to another task, which is identifying the types of DFUs from images. In addition, the performances of the CNN models depend heavily on the training procedure. To implement transfer learning, we used the original architectures for the different CNN models used in this study, removing the top layer of each model and using ImageNet for the initial weights. This excludes the final dense layer, specifically the fully connected (dense) layer that is responsible for mapping the 1280-dimensional feature vector from the penultimate layer into predictions for the 1000 ImageNet classes. This adjustment retains the convolutional base of the models, which outputs feature maps, and is particularly advantageous for transfer learning scenarios. Moreover, we freeze the weights of a specific layer by setting layer.trainable = False to keep the weights of that particular layer fixed during the training process.

To deliver superior results, the hyperparameter tuning of the CNN models was performed during this phase to establish the best settings of the CNN pre-trained models in ischemia classification and infection classification.

Table 4 illustrates the hyperparameter values that were used with all the models.

### 3.5. Modified Architectures of CNNs (The Proposed Head Model)

**Figure 4:**
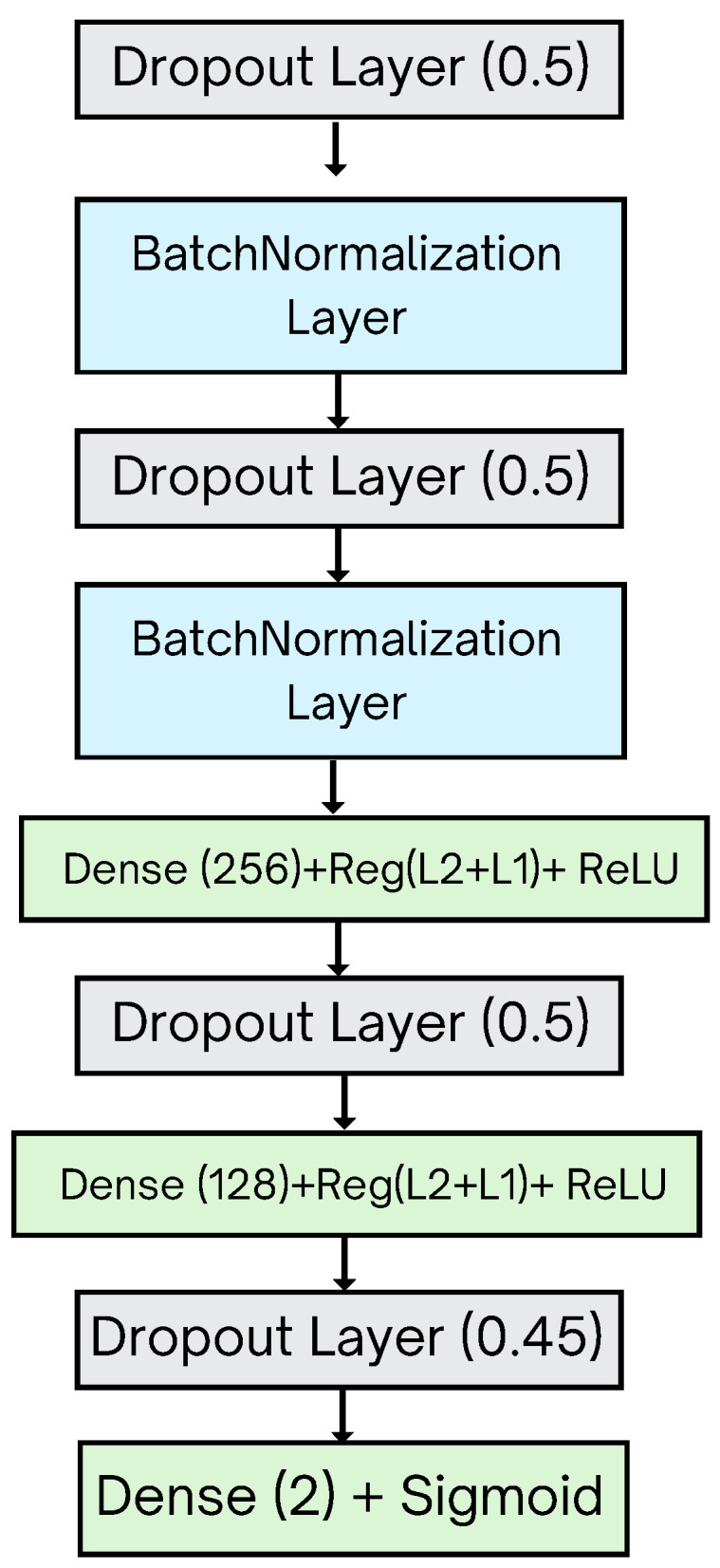
Proposed head model structure.

After employing various pre-existing CNN models to identify the most effective one for categorizing DFU images for both infections and ischemia, we compared the performances of the pre-trained models after combining them with the proposed head model to address the overfitting issue and improve the results. The analysis of the loss graphs for the pre-trained CNN models in the first stage indicated that while the training loss decreased steadily, the validation loss initially dropped but then started to rise, indicating an overfitting issue. Moreover, the accuracy graphs showed a gap between the training and validation accuracy, which also indicates an overfitting issue. This learning problem prompted the development of a new head model positioned at the end of CNN models to address the overfitting issue and improve performance. Figure 4 illustrates the structure of the proposed head model.

The structure of the head model was refined through multiple experiments involving different combinations of layer types and values to find an effective model structure for preventing overfitting. The selected model structure showed optimal fitting learning curves in both loss graphs and accuracy graphs of CNN models, with a noticeable decrease in both training and validation losses. In addition, the training and validation accuracy are increased, and the gap between them is decreased. The proposed head model design effectively tackled overfitting issues and improved performance outcomes by incorporating multiple layers arranged in a specific order, as shown in Table 5.

A dropout layer is used to prevent overfitting and enhance the generalization ability of the CNN models. A dropout rate of 0.5 signifies that 50% of input units (neurons) will be deleted during training to reduce the complexity of the CNN models. A BatchNormalization layer is used to provide better generalization performance of the CNN models.

The dropout and batch normalization layers were carefully chosen and arranged through multiple experiments to determine the most effective configuration that yields optimal results. Similarly, the number of dense layers and their units were extensively explored to achieve the best outcome. In addition, a combination of regularization techniques including kernel regularizer, L1 and L2 regularization, activity regularization, and bias regularization is employed to address overfitting issues and manage the CNN model complexity. The values for these regularization techniques were selected based on various experiments to identify the most suitable settings.

In the dense layers, the model employs the ReLU activation function, which has been found to be the most suitable activation function after testing various activation functions. The final layer contains a dense layer with 2 units, facilitating binary classification output with a sigmoid activation function, which is well suited for binary classification tasks. The proposed approach will be used for ischemia classification to perform the binary classification of ischemia (ischemia or non-ischemia). Similarly, the same proposed approach will be used for infection classification to perform the binary classification of infection (infection or non-infection).

### 3.6. Feeding the Model Results to Machine Learning Classifiers

The results of the modified architectures of CNN models, after the proposed head model is added, are fed to different ML algorithms to enhance the overall results of the modified architectures of CNN models in both infection classification and ischemia classification. To identify the best ML classifiers that can be used with each CNN model with the proposed head model, the LazyClassifier function was utilized. LazyClassifier is a function included in the LazyPredict library that ranks the performances of various ML classifiers based on their results from best to worst. LazyClassifier is used for predicting binary variables. During this phase, the ML classifier that provided the best results compared to other ML classifiers was chosen to report its results.

## 4. Experimental Results and Discussion

This section presents and analyzes the findings of this research, involving both the initial phase results and the outcomes at subsequent phases. Each subsection includes tables that outline the performance of each CNN model using various assessment measures, such as accuracy, precision, sensitivity, specificity, F1 score, area under the curve (AUC), and processing time for each DFU state, ischemia classification, and infection classification. In addition, ROC curves illustrating the performance of all the CNN models are included for all phases of ischemia classification and infection classification. Moreover, this section features graphs depicting the loss curve and accuracy curve of the CNN models before and after integrating the proposed head model to demonstrate notable enhancements in learning curves for both ischemia classification and infection classification. A significant improvement is observed in the learning trajectory of these models after incorporating the proposed head model.

### 4.1. Performance Evaluation Metrics

The output of the proposed approach is binary classification. The proposed binary classification approach was applied to infection images (infection, non-infection) and ischemia images (ischemia, non-ischemia). Various assessment measures are available for computing data classification outcomes. The computation depends on the true positive (TP) and true negative (TN) values, indicating the accurate classification of positive and negative cases, as well as false positive (FP) and false negative (FN) values, denoting misclassified negative and positive cases [24].

(1) $$\mathrm{Precision}=\frac{\mathrm{TP}}{\mathrm{TP}\;+\;\mathrm{FP}}$$

(2) $$\;\mathrm{Recall}\;\left(\mathrm{Sensitivity}\right)=\frac{\mathrm{TP}}{(\mathrm{TP}\;+\;\mathrm{FN})}$$

(3) $$\mathrm{Specificity}=\frac{\mathrm{TN}}{\mathrm{TN}\;+\;\mathrm{FP}}$$

(4) $$F1\text{-}\mathrm{Measure}=\frac{2\cdot \mathrm{TP}}{2\cdot \mathrm{TP}\;+\;\mathrm{FP}\;+\;\mathrm{FN}}$$

(5) $$\mathrm{Accuracy}=\frac{\mathrm{TP}\;+\;\mathrm{TN}}{\mathrm{TP}\;+\;\mathrm{TN}\;+\;\mathrm{FP}\;+\;\mathrm{FN}}$$

Moreover, the area under curve (AUC) is a measure of the ability of the classifier to differentiate between two classes:

(6) $$\mathrm{AUC}=\frac{S_{p}-n_{p}\left(n_{n}+1\right)/2}{n_{p}n_{n}}$$

where $S_{p}$ is the sum of the all positive example, $n_{p}$ is the number of positive example, and $n_{n}$ is the number of negative example.

### 4.2. Results of Competitor Models

Table 6 presents the results of CNN models (EfficientNetB0, DenseNet121, ResNet101, VGG16, InceptionV3, MobileNetV2, and InceptionResNetV2) in ischemia classification by accuracy, precision, sensitivity, specificity, F1 score, AUC, and time (S). Additionally, Table 7 presents the results of CNN models in infection classification using the same evaluation metrics. Both Table 6 and Table 7 indicate that the EfficientNetB0 model outperformed other CNN models in both ischemia and infection classifications.

The EfficientNetB0 model achieved 0.947 accuracy, 0.950 precision, 0.943 sensitivity, 0.950 specificity, 0.947 F1 score, and 0.947 AUC in ischemia classification. For infection classification, the EfficientNetB0 model obtained 0.904 accuracy, 0.886 precision, 0.926 sensitivity, 0.881 specificity, 0.906 F1 score, and 0.904 AUC.

**Figure 5:**
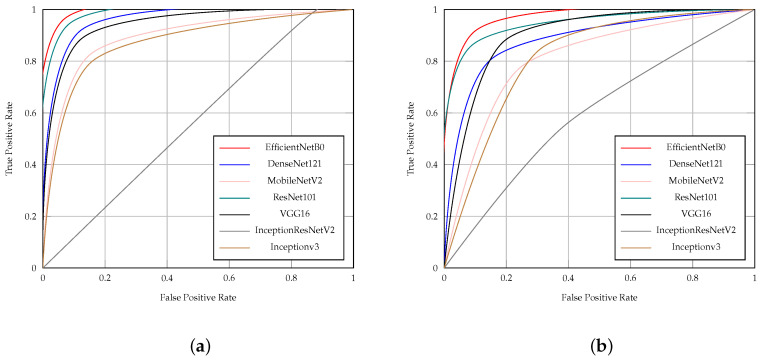
ROC curves of the CNN pre-trained models for (**a**) ischemia and (**b**) infection classification.

Figure 5a depicts the ROC curves of the CNN models in classifying ischemia, while Figure 5b shows the ROC curves of the CNN models in classifying infection. The EfficientNetB0 CNN model demonstrated superior performance in both ischemia classification and infection classification, evident from its closer proximity to the upper left corner of the ROC curve. This indicates that the true positive (TP) rate of EfficientNetB0 is higher than that of other models, while its false positive (FP) rate is lower.

### 4.3. Results of Modified Architectures of CNNs

Table 8 shows the outcomes of the CNN models with the proposed head model for ischemia classification, whereas Table 9 presents the findings of CNN models utilizing the proposed head model for infection classification. The highest performance was observed in both ischemia and infection classifications when employing the EfficientNetB0 model in combination with the proposed head model, surpassing other models used alongside this approach. The EfficientNetB0 model combined with the proposed head model produced encouraging outcomes with 0.965 for accuracy, 0.959 for precision, 0.971 for sensitivity, 0.958 for specificity, 0.965 for the F1 score, and 0.965 for the AUC in the ischemia classification. Furthermore, the EfficientNetB0 model with the proposed head model produced impressive results in infection classification with 0.919 for accuracy, 0.951 for precision, 0.883 for sensitivity, 0.954 for specificity, 0.916 for F1 score, and 0.919 for the AUC.

After integrating the proposed head model into the CNN models, the time metric shows a slight increase after incorporating the head model in most of the models as shown in Table 6, Table 7, Table 8 and Table 9. This is attributed to the increased complexity resulting from additional layers and regularization techniques, including dropout, L1, and L2, which have been introduced to address overfitting issues and enhance generalizability with unseen data.

Moreover, there was a notable improvement in ischemia classification for EfficientNetB0, ResNet101, DenseNet121, and InceptionResNetV2. In addition, adding the proposed head model led to enhanced results for EfficientNetB0, ResNet101, DenseNet121, VGG16,and InceptionResnetV2 in infection classification. Overall, incorporating the proposed head model resulted in increased accuracy across all CNN models by addressing overfitting issues commonly encountered by these models. The CNN models’ performance showed a significant improvement with the addition of the proposed head model, as depicted in Figures 13–16. This enhancement is evident in both the loss curve and accuracy curve for ischemia classification and infection classification. Having models that exhibit an ideal learning curve is of greater importance compared to having models that offer higher accuracy but suffer from overfitting. The addition of the proposed head model significantly enhances the learning curve, effectively addressing the issue of overfitting. As a result, the models demonstrate improved generalizability and accuracy due to the appropriate complexity structure provided by the proposed head model, making them more dependable for practical use.

**Figure 6:**
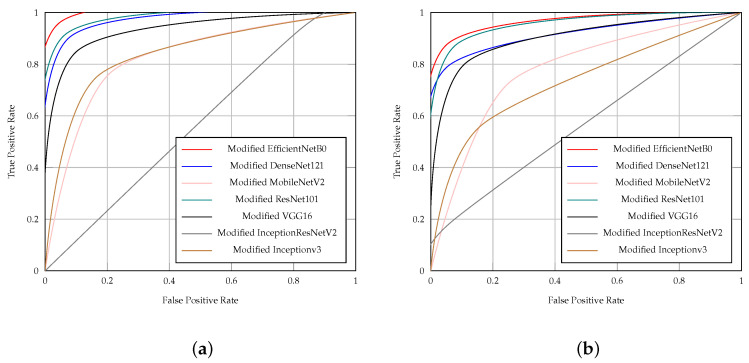
ROC curves of the CNN pre-trained models with the proposed head model for (**a**) ischemia and (**b**) infection classification.

Figure 6a shows the ROC curves of the CNN models with the proposed head model in ischemia classification. Furthermore, Figure 6b illustrates the ROC curves of the CNN models with the proposed head model in infection classification. The ROC curves of the models in Figure 6 show that the EfficientNetB0 CNN model with the proposed head model outperforms the other models, which are DenseNet121, ResNet101, VGG16, InceptionV3, MobileNetV2, and InceptionResNetV2, all with the proposed head model, in both ischemia classification and infection classification.

### 4.4. Results of Effect of Augmentation Process

**Figure 7:**
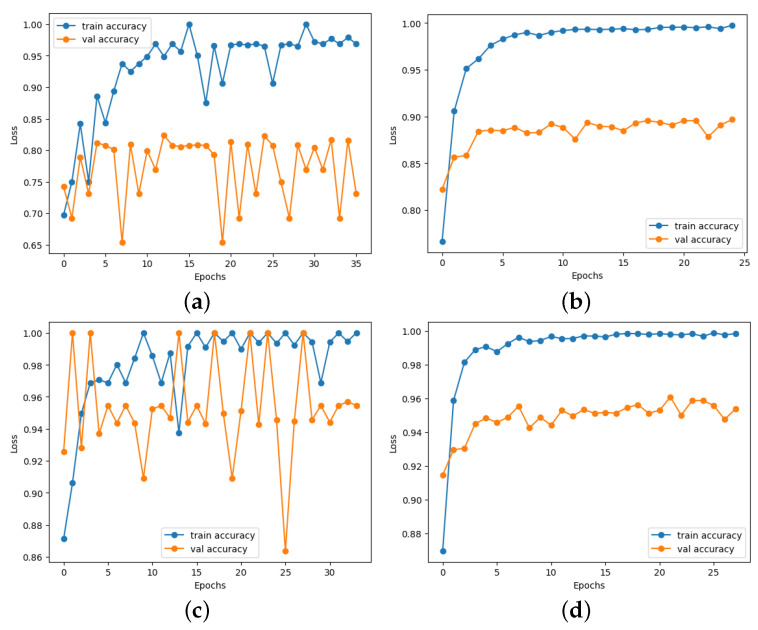
Results of the accuracy of EfficientNetB0 in infection classification (top row) and ischemia classification (lower row) on the DFU-Part (B) dataset: (**a**,**c**) before augmentation and (**b**,**d**) after augmentation.

The differences in accuracy between the augmented and non-augmented datasets for both ischemia and infection classification are proven for all competitors and shown, e.g., in Figure 7 for EfficientNetB0. This empirically demonstrates that the augmented dataset significantly enhances the accuracy of the classification models. Given that the visual data we worked with have high inherent variability, it is necessary for the models to learn in a robust manner—learning generalized features rather than just the memorization of specific instances.

### 4.5. Results of Feeding Model Results to Machine Learning Classifiers

Table 10 presents the results of feeding the result of each CNN model with the proposed head model to the selected ML classifier in ischemia classification. On the other hand, Table 11 shows the results of each CNN model with the proposed head model after feeding the result of each model to the selected ML classifier in infection classification. The ML classifier that was used with each CNN model with the proposed head model was selected based on the best performances of different ML classifiers in enhancing the overall results of each CNN model with the proposed head model compared to other ML classifiers. For the time metric, Table 10 and Table 11 include the training time of the CNN models with the proposed head model plus the time that the ML classifier took to produce the final result after feeding the result of the CNN models with the proposed head model to the selected ML classifier.

Feeding the result of the EfficientNetB0 model with the proposed head model to the LogisticRegression ML classifier provided the highest results in ischemia classification compared to the results of the other CNN model with the proposed head model after feeding them with the selected ML classifier. The results of the EfficientNetB0 model with the proposed head model after it is fed to the LogisticRegression ML classifier in ischemia classification are 0.967, 0.967, 0.968, 0.967, 0.967, and 0.967 for the accuracy, the precision, the sensitivity, the specificity, the F1 score, and the AUC, respectively. The highest result in infection classification is achieved after feeding the result of the EfficientNetB0 model with the proposed head model to the AdaBoostClassifier ML classifier with 0.927 for accuracy, 0.934 for precision, 0.919 for sensitivity, 0.936 for specificity, 0.927 for the F1 score, and 0.927 for the AUC, respectively.

**Figure 8:**
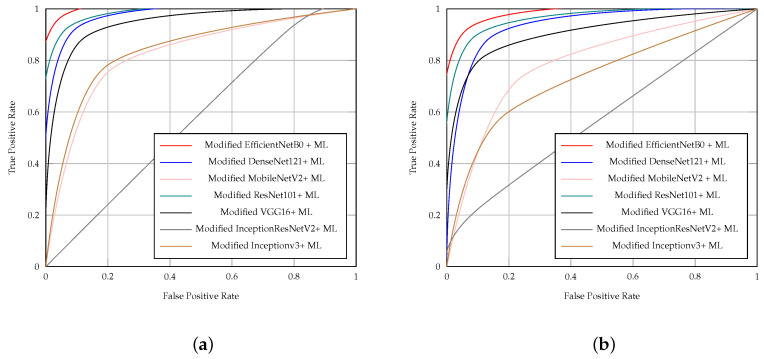
ROC curves of the CNN pre-trained models with the proposed head model after feeding the results to ML classifier for (**a**) ischemia and (**b**) infection classification.

Figure 8a illustrates the ROC curves of the models after feeding the results of the CNN models with the proposed head model to the selected ML classifier in ischemia classification. In regards to infection classification, Figure 8b illustrates the ROC curves of the CNN models with the proposed head model after feeding the results of the models to the selected ML classifier. The ROC curves of the models in Figure 8a show that feeding the results of the EfficientNetB0 CNN model with the proposed head model to the LogisticRegression ML classifier outperforms the performance of the other CNN model with the proposed head model after feeding them with the selected ML classifier in ischemia classification. Furthermore, Figure 8b shows that feeding the results of the EfficientNetB0 CNN model with the proposed head model to the AdaBoostClassifier ML classifier outperforms the performance of the other CNN model with the proposed head model after feeding them to the selected ML classifier in infection classification.

**Figure 9:**
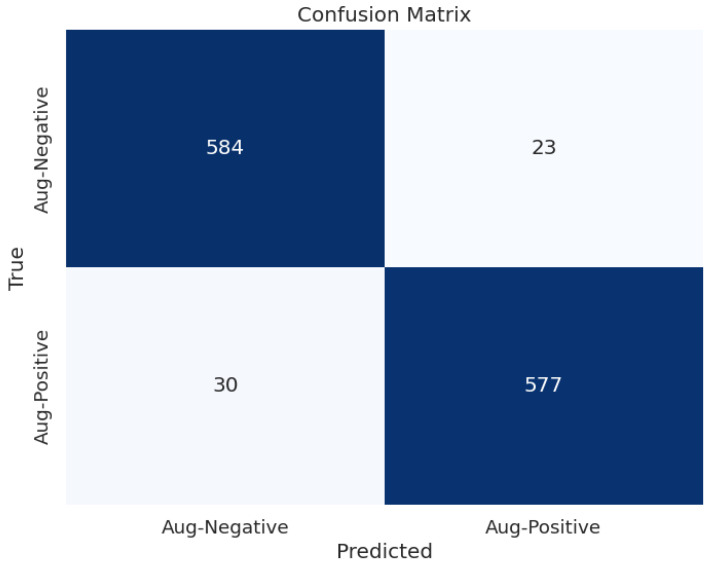
Confusion matrix of EfficientNetB0 with the proposed head model after feeding the results of them to the LogisticRegression classifier for ischemia classification.

**Figure 10:**
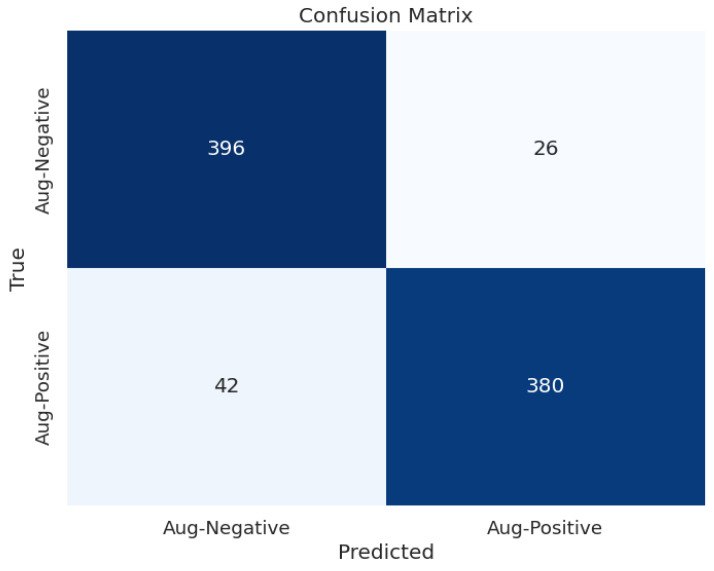
Confusion matrix of EfficientNetB0 with the proposed head model after feeding the results of them to the AdaBoostClassifier classifier for infection classification.

Figure 9 shows the confusion matrix of the selected approach that provides the best results in ischemia classification, which is the EfficientNetB0 with the proposed head model after feeding the results to the LogisticRegression classifier. Moreover, Figure 10 illustrates the confusion matrix of the selected approach that provides the highest results in infection classification, which is EfficientNetB0 with the proposed head model after feeding the results to the AdaBoostClassifier classifier.

**Figure 11:**
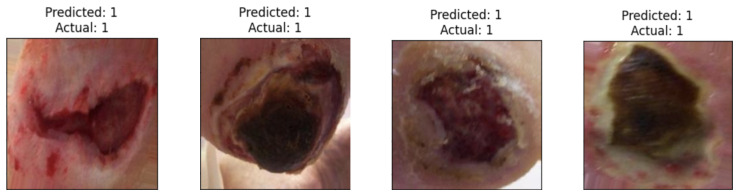
Sample of correctly classified images from DFU-Part (B) dataset of ischemia for ischemia classification.

**Figure 12:**
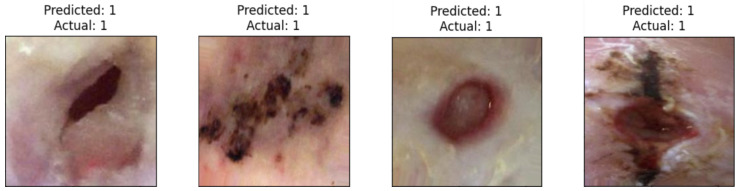
Sample of correctly classified images from DFU-Part (B) dataset of infection for infection classification.

Figure 11 illustrates instances of ischemia that have been correctly categorized by the selected approach that provides the highest results in ischemia classification, which is EfficientNetB0 with the proposed head model after feeding the results to the LogisticRegression classifier. Figure 12 presents images of infections accurately classified by the selected approach that provides the best results in infection classification, which is EfficientNetB0 with the proposed head model after feeding the results to the AdaBoostClassifier classifier.

### 4.6. Loss and Accuracy Curve Graphs

**Figure 13:**
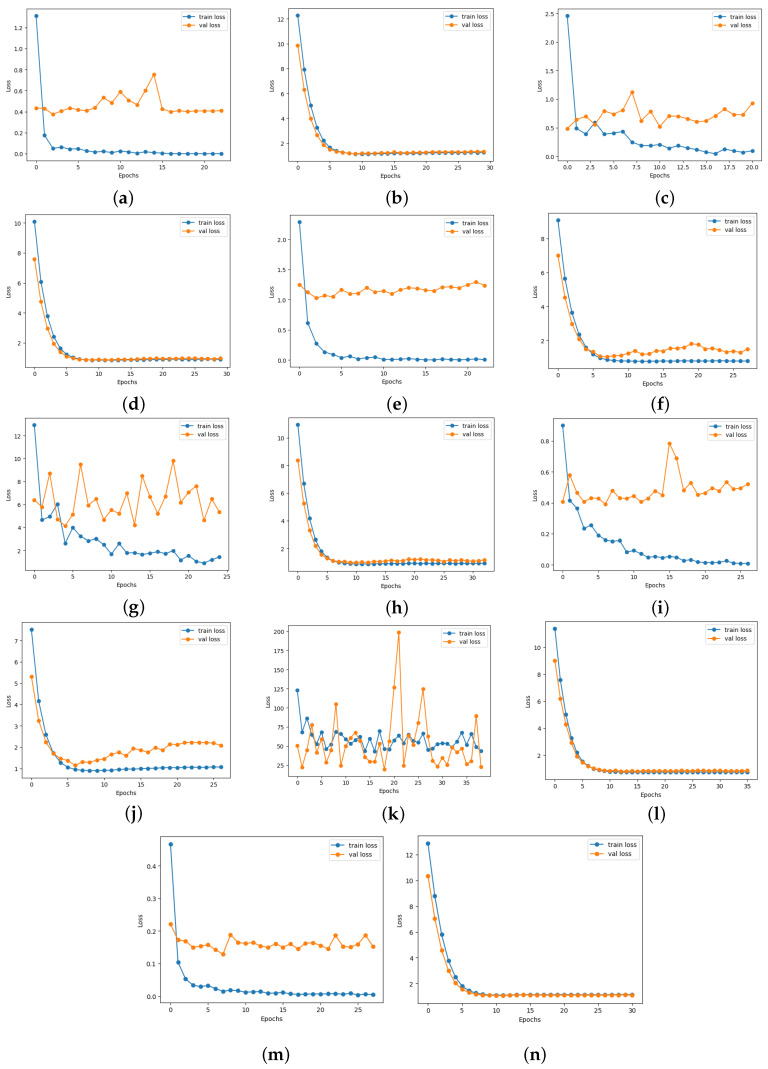
Training and validation loss curves of each pre-trained model before and after adding the proposed head model in ischemia classification (**a**) ResNet101, (**b**) Modified ResNet101, (**c**) DenseNet121, (**d**) Modified DenseNet121, (**e**) VGG16, (**f**) Modified VGG16, (**g**) InceptionV3, (**h**) Modified InceptionV3, (**i**) MobileNetV2, (**j**) Modified MobileNetV2, (**k**) InceptionResNetV2, (**l**) Modified InceptionResNetV2, (**m**) EfficientNetB0, and (**n**) Modified EfficientNetB0.

**Figure 14:**
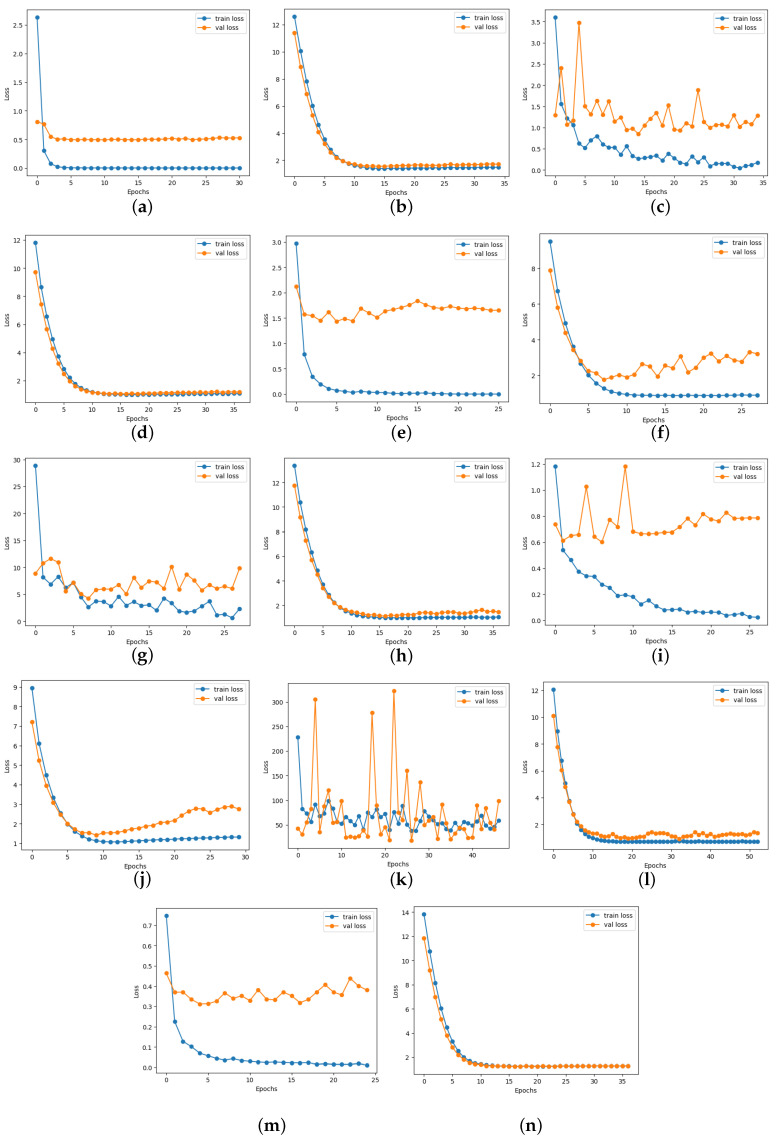
Training and validation loss curves of each pre-trained model before and after adding the proposed head model in infection classification(**a**) ResNet101, (**b**) Modified ResNet101, (**c**) DenseNet121, (**d**) Modified DenseNet121, (**e**) VGG16, (**f**) Modified VGG16, (**g**) InceptionV3, (**h**) Modified InceptionV3, (**i**) MobileNetV2, (**j**) Modified MobileNetV2, (**k**) InceptionResNetV2, (**l**) Modified InceptionResNetV2, (**m**) EfficientNetB0, and (**n**) Modified EfficientNetB0.

**Figure 15:**
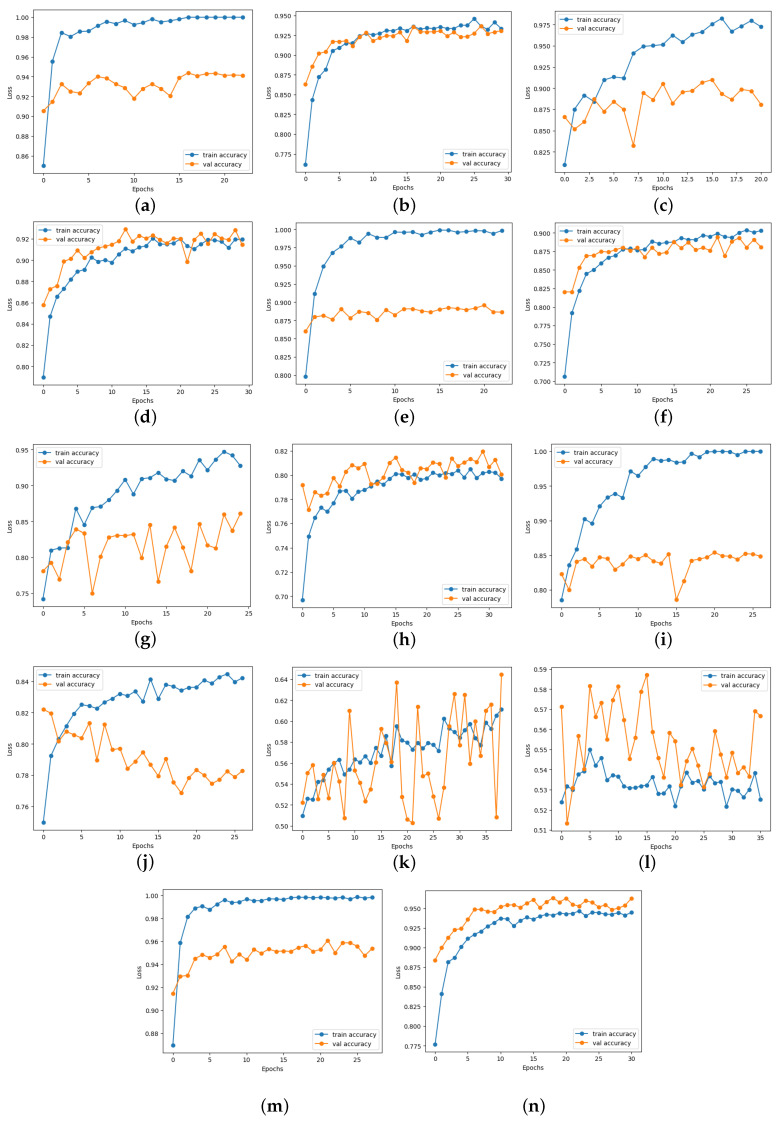
Training and validation accuracy curves of each pre-trained model before and after adding the proposed head model in ischemia classification (**a**) ResNet101, (**b**) Modified ResNet101, (**c**) DenseNet121, (**d**) Modified DenseNet121, (**e**) VGG16, (**f**) Modified VGG16, (**g**) InceptionV3, (**h**) Modified InceptionV3, (**i**) MobileNetV2, (**j**) Modified MobileNetV2, (**k**) InceptionResNetV2, (**l**) Modified InceptionResNetV2, (**m**) EfficientNetB0, and (**n**) Modified EfficientNetB0.

**Figure 16:**
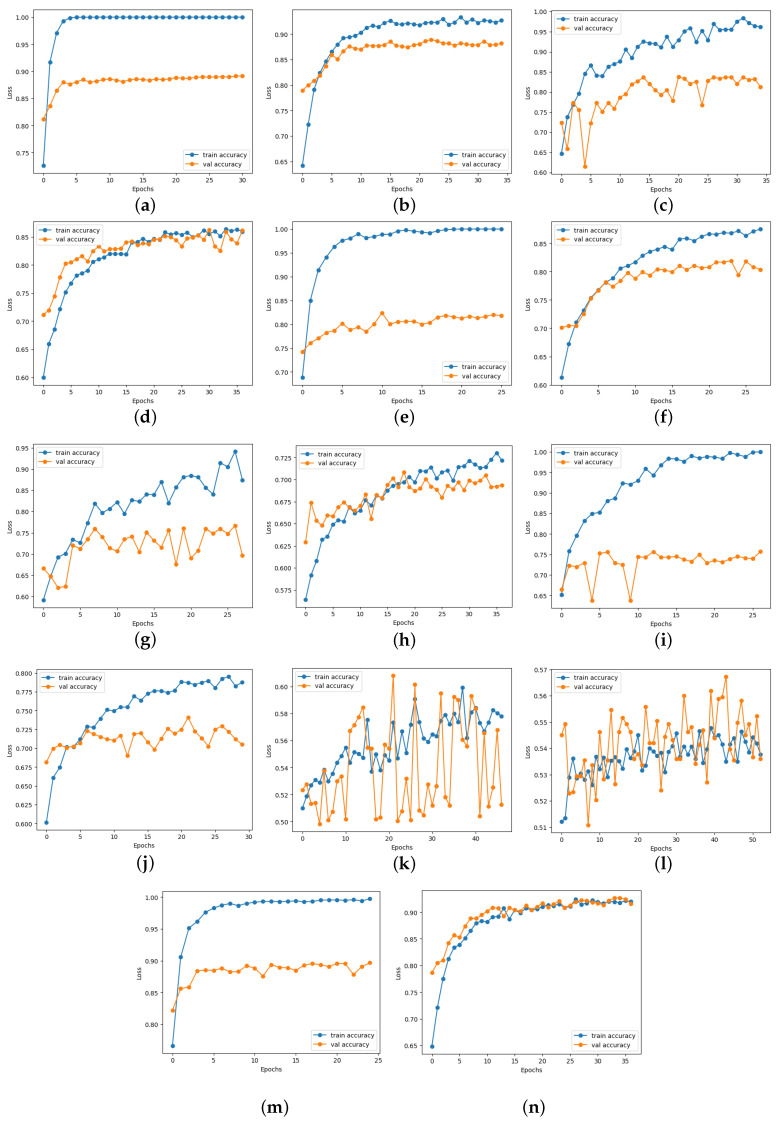
Training and validation accuracy curves of each pre-trained model before and after adding the proposed head model in infection classification (**a**) ResNet101, (**b**) Modified ResNet101, (**c**) DenseNet121, (**d**) Modified DenseNet121, (**e**) VGG16, (**f**) Modified VGG16, (**g**) InceptionV3, (**h**) Modified InceptionV3, (**i**) MobileNetV2, (**j**) Modified MobileNetV2, (**k**) InceptionResNetV2, (**l**) Modified InceptionResNetV2, (**m**) EfficientNetB0, and (**n**) Modified EfficientNetB0.

Figure 13 depicts the loss curve graphs of all CNN models before and after integrating the suggested head model for ischemia classification, while Figure 14 illustrates the loss curve graphs of all CNN models before and after incorporating the proposed head model for infection classification. It is evident from the loss curves in Figure 13 for ischemia classification and Figure 14 for infection classification that the learning curve of CNN models reaches an optimal state once the proposed head model is integrated. Before incorporating the head models, it was evident from the loss curve that the CNN models were experiencing challenges with overfitting. The training loss plot showed a continuous decline, while the validation loss plot decreased initially and then began to increase, signaling an overfitting issue. However, following the addition of the head model to the CNN models, both training and validation losses exhibited a decrease, indicating optimal learning for these models. Figure 15 shows the accuracy trend of the CNN models before and after adding the head model for ischemia classification, while Figure 16 displays the corresponding graphs for infection classification. These visuals demonstrate an increase in validation accuracy following the integration of the proposed head model, as well as a decrease in the gap between train accuracy and validation accuracy. These results indicate improved model precision and overall performance enhancement.

### 4.7. Discussion

This study focused on classifying DFU images into binary classifications of each DFU state, ischemia and infection, to avoid DFU misdiagnosis due to the similarities of the two states of DFUs. The proposed approach in this study will aid in the development of more efficient automatic DFU assessment intelligent telemedicine systems that assist in diagnosing DFUs correctly and more promptly, which helps in providing effective treatment to prevent the worsening of DFUs and reduce the risk of negative consequences.

This study shows that the EfficientNetB0 pre-trained model provides the best results in both ischemia classification and infection classification compared to the DenseNet121, ResNet101, VGG16, 9 InceptionV3, MobileNetV2, and InceptionResNetV2 pre-trained models. Moreover, this study shows the significance of adding the proposed head model to the pre-trained CNN models in improving the performances of the models by overcoming the overfitting problem, which clearly shows in the improvement of the learning curve of the models.

In addition, solving the overfitting problem by adding the proposed head model to the CNN models makes the CNN models more accurate, reliable, and generalizable with unseen data. Additionally, the results of most of the CNN models are increased after adding the proposed head model with all the evaluation metrics. Furthermore, in this study, the results of each CNN model with the proposed head model were fed to different ML classifiers to select the best classifier that can be used with each model to enhance the overall results of the proposed approach.

The highest achieved result in this study for ischemia classification was achieved by the EfficientNetB0 pre-trained CNN model with the proposed head model after feeding its results to the LogisticRegression ML classifier with 0.967, 0.967, 0.968, 0.967, 0.967, and 0.967 for the accuracy, the precision, the sensitivity, the specificity, the F1 score, and the AUC, respectively. For the infection classification, the highest result was achieved by the EfficientNetB0 pre-trained model with the proposed head model after feeding its results to the AdaBoostClassifier ML classifier with 0.927 for the accuracy, 0.934 for the precision, 0.919 for the sensitivity, 0.936 for the specificity, 0.927 for the F1 score, and 0.927 for the AUC (Table 12).

According to the literature, the results achieved in this study are higher than the achieved results of previous studies that used pre-trained CNN models in classifying DFU images, as a binary classification of each ischemia classification and infection classification. Goyal et al. [17], used three pre-trained models (Inception-V3, InceptionResNetV2, and ResNet50) in an ensemble approach and achieved 90% for the accuracy in ischemia classification and 73% for the accuracy in infection classification. Moreover, Al-Garaawi et al. [20] used a pre-trained GoogLeNet CNN model and achieved 92% accuracy in ischemia classification and 73% accuracy in infection classification. The accuracy rates achieved in this study were higher than those of the aforementioned studies. This study achieved 97% and 93% for the accuracy in ischemia classification and infection classification, respectively.

Table 13 and Table 14 show comparisons of the pre-trained CNN model results on ischemia classification and infection classification in each stage of the proposed approach based on accuracy, before adding the proposed head model, after adding the proposed head model, and, finally, after feeding the results of the CNN pre-trained model with the proposed head model to the selected ML classifier. It is evident from the result comparison that the results of most of the CNN models improved after adding the proposed head model. However, even if the results of some of the CNN models are not increased, the models become more accurate as a result of solving the overfitting problem. This increased accuracy makes the models more reliable and ready to be implemented in a clinical environment. In addition, the results of the various CNN models with the proposed head model are enhanced after the results are fed to the selected ML classifier.

## 5. Conclusions

DFU prevalence is increasing and affecting a larger number of people every day. The early detection of DFU enables prompter and more effective intervention. The timely identification and treatment of DFUs can increase survival rates and decrease mortality. However, existing clinical approaches for recognizing DFUs are susceptible to human error due to subjectivity and insufficient expertise among healthcare professionals. As a result, there is a need for more dependable, precise, and user-friendly solutions to support physicians in this area. In this study, we introduced a proposed model to address overfitting and improve the performance of pre-trained CNN models. The effectiveness of pre-trained fine-tuned models such as EfficientNetB0, ResNet101, DenseNet121, VGG16, IInceptionV3, MobileNetV2, and InceptionResNetV2 was examined to perform the binary classification of ischemia/non-ischemia and infection/non-infection states before and after adding the proposed model, involving ML approaches and hyperparameter tuning. The results show that the proposed model effectively addressed overfitting issues while enhancing results across different ML classifiers for CNN models with the proposed head model. We conclude that the EfficientNetB0 model provides the best results compared to other CNN pre-trained models, which are ResNet101, DenseNet121, etc. in both ischemia classification and infection classification. Furthermore, this study showed that the EfficientNetB0 model with the proposed head model and after feeding the results to the LogisticRegression classifier achieved the highest result in ischemia classification. Additionally, for infection classification, the EfficientNetB0 model with the proposed head model and feeding the results to AdaBoostClassifier classifier achieved the highest result.

For future work, the proposed approach can be used with a larger dataset when a larger dataset for ischemia classification and infection classification of the DFU images becomes available. Moreover, the proposed approach can be used with different diseases to classify them based on the images with different classes. Moreover, this proposed approach can be used for different diabetes complications such as mouth ulcers caused by rosuvastatin [25] or monitoring the development or regression of diabetic wounds during laboratory experiments [26]. Furthermore, by providing a more comprehensive understanding of the abilities of various CNNs to classify DFU images, this work helps to clarify the significance of the backbone’s capability in developing a lightweight model to handle such diagnoses in real time.

## Figures and Tables

**Table 1:** Studies that implemented binary classification for infection and ischemia.

Author [ref.]	Year	Model	Dataset	Evaluation Criteria	Result
Goyal et al. [17]	2020	Ensemble CNN (Inception-V3, InceptionResNetV2, and ResNet50) with SVM classifier	DFU-Part (B)	Accuracy	Ischemia: 90%, infection: 73%
Amin et al. [18]	2020	Proposed CNN	DFU-Part (B)	Accuracy	Ischemia: 0.976, infection: 0.996
Al-Garaawi et al. [19]	2022	Proposed CNN DFU-RGB-TEX-NET	DFU-Part (A) and DFU-Part (B)	Accuracy	Ischemia: 99%, Infection: 74%
Al-Garaawi et al. [20]	2022	GoogLNet CNN with RF	DFU-Part (A) and DFU-Part (B)	Accuracy	Ischemia: 92% Infection: 73%
Xu et al. [21]	2022	Transformer-based DeiT model with class knowledge banks (CKBs)	DFU-Part (B)	Accuracy	Ischemia: 90.9%, infection: 78%
Das et al. [22]	2022	ResKNet	DFU-Part (B)	Accuracy	Res4Net for ischemia: 97.8%, Res7Net for infection: 80%

**Table 2:** Summary of the number of images in the DFU-Part (B) dataset for each class before and after natural data augmentation and after our augmentation process.

Classification Type	Class	No of Images	No. of Natural Augmented Images	No. of Augmented Images (Ours)
Ischemia Classification	Ischemia	1249	4935	6062
	Non-ischemia	210	4935	6062
Infection Classification	Infection	628	2945	4212
	Non-infection	831	2945	4212

**Table 3:** Parameter details used for data augmentation.

Operation Name	Value
Rotation	$30^{\circ }$
Flip	Horizontal/Vertical

**Table 4:** Hyperparameter values.

Parameter Name	Value
Optimizer	Adamax
Learning Rate	0.001
Patience of the EarlyStopping	20
Batch Size	32
Epochs	100

**Table 5:** Details of proposed head model structure.

Layer Name	Values
Dropout Layer	0.5 Rate
BatchNormalization	Default Values
Dropout Layer	0.5 Rate
BatchNormalization	Default Values
Dense Layer	256 Units + kernel reg = l2(l = 0.016) + activity reg = l1(0.006) + bias reg = l1(0.006) + ReLU
Dropout Layer	0.5 Rate
Dense Layer	128 Units + kernel reg = l2(l = 0.016) + activity reg = l1(0.006) + bias reg = l1(0.006) + ReLU
Dropout Layer	0.45 Rate
Dense Layer	2 Units + Sigmoid

**Table 6:** Ischemia classification results of CNN pre-trained models.

Name of the Pre-Trained CNN Modified Model	Accuracy	Precision	Sensitivity	Specificity	F1 Score	AUC	Time (S)
EfficientNetB0	**0.947**	**0.950**	**0.943**	**0.950**	**0.947**	**0.947**	1895
ResNet101	0.925	0.912	0.940	0.909	0.926	0.925	1688
DenseNet121	0.899	0.883	0.920	0.878	0.901	0.899	1135
VGG16	0.886	0.892	0.878	0.894	0.885	0.886	1632
InceptionV3	0.819	0.766	0.919	0.719	0.835	0.819	1105
MobileNetV2	0.832	0.809	0.869	0.795	0.838	0.832	880
InceptionResNetV2	0.560	0.534	0.950	0.21	0.684	0.560	2163

Bold values indicate the highest values.

**Table 7:** Infection classification results of CNN pre-trained models.

Name of the Pre-Trained CNN Modified Model	Accuracy	Precision	Sensitivity	Specificity	F1 Score	AUC	Time (S)
EfficientNetB0	**0.904**	**0.886**	**0.926**	**0.881**	**0.906**	**0.904**	831
ResNet101	0.896	0.917	0.872	0.921	0.894	0.896	2109
DenseNet121	0.829	0.814	0.853	0.805	0.833	0.829	653
VGG16	0.827	0.822	0.834	0.819	0.828	0.827	1010
InceptionV3	0.763	0.824	0.668	0.857	0.738	0.763	725
MobileNetV2	0.747	0.717	0.817	0.677	0.764	0.747	747
InceptionResNetV2	0.535	0.522	0.810	0.260	0.635	0.535	1193

Bold values indicate the highest values.

**Table 8:** Ischemia classification results of CNN pre-trained models with the proposed head model.

Name of the Pre-Trained CNN Modified Model	Accuracy	Precision	Sensitivity	Specificity	F1 Score	AUC	Time (S)
Modified EfficientNetB0	**0.965 **	**0.959 **	**0.971 **	**0.958 **	**0.965 **	**0.965 **	1263
Modified ResNet101	0.933	0.939	0.925	0.940	0.932	0.933	2383
Modified DenseNet121	0.902	0.922	0.879	0.925	0.900	0.902	1226
Modified VGG16	0.878	0.897	0.853	0.902	0.875	0.878	1581
Modified InceptionV3	0.775	0.854	0.665	0.886	0.748	0.775	1173
Modified MobileNetV2	0.785	0.766	0.818	0.751	0.792	0.785	1046
Modified InceptionResNetV2	0.638	0.596	0.851	0.425	0.701	0.638	2540

Bold values indicate the highest values.

**Table 9:** Infection classification results of CNN pre-trained models with the proposed head model.

Name of the Pre-Trained CNN Modified Model	Accuracy	Precision	Sensitivity	Specificity	F1 Score	AUC	Time (S)
Modified EfficientNetB0	**0.919 **	**0.951 **	**0.883 **	**0.954 **	**0.916 **	**0.919 **	1209
Modified ResNet101	0.899	0.918	0.876	0.921	0.896	0.899	2311
Modified DenseNet121	0.868	0.935	0.791	0.945	0.857	0.868	981
Modified VGG16	0.847	0.915	0.765	0.928	0.833	0.847	1143
Modified InceptionV3	0.707	0.761	0.604	0.810	0.673	0.707	1241
Modified MobileNetV2	0.738	0.741	0.732	0.744	0.736	0.738	841
Modified InceptionResNetV2	0.561	0.732	0.194	0.928	0.307	0.561	2740

Bold values indicate the highest values.

**Table 10:** Ischemia classification results of CNN pre-trained models with the proposed head model after feeding the results into ML classifier.

Name of the Pre-Trained Model + Proposed Head Model + ML	Accuracy	Precision	Sensitivity	Specificity	F1 Score	AUC	Time (S)
(EfficientNetB0 + Proposed Model) + LogisticRegression	**0.967**	**0.967 **	**0.968 **	**0.967 **	**0.967 **	**0.967 **	1263 + 0.06
(ResNet101 + Proposed Model) + XGBClassifier	0.935	0.940	0.930	0.940	0.935	0.935	2383 + 0.23
(DenseNet121 + Proposed Model ) + KNeighborsClassifier	0.911	0.911	0.912	0.911	0.911	0.911	1226 + 0.07
(VGG16 + Proposed Model) + KNeighborsClassifier	0.882	0.881	0.883	0.881	0.882	0.882	1581 + 0.07
(InceptionV3 + Proposed Model) + XGBClassifier	0.777	0.757	0.817	0.738	0.786	0.777	1173 + 0.90
(MobileNetV2 + Proposed Model) + AdaBoostClassifier	0.784	0.773	0.803	0.764	0.788	0.784	1046 + 0.64
(InceptionResNetV2 + Proposed Model) + AdaBoostClassifier	0.652	0.616	0.805	0.499	0.698	0.652	2540 + 0.25

Bold values indicate the highest values.

**Table 11:** Infection classification results of CNN pre-trained models with the proposed head model after feeding the results into ML classifier.

Name of the Pre-Trained Model + Proposed Head Model + ML	Accuracy	Precision	Sensitivity	Specificity	F1 Score	AUC	Time (S)
(EfficientNetB0 + Proposed Model) + AdaBoostClassifier	**0.927 **	**0.934 **	**0.919 **	**0.936 **	**0.927 **	**0.927 **	1209 + 0.23
(ResNet101 + Proposed Model) + LogisticRegression	0.900	0.918	0.879	0.921	0.898	0.900	2311 + 0.12
(DenseNet121 + Proposed Model) + XGBClassifier	0.874	0.867	0.883	0.864	0.875	0.874	981 + 0.08
(VGG16 + Proposed Model) + LogisticRegression	0.863	0.909	0.808	0.919	0.855	0.863	1143 + 0.11
(InceptionV3 + Proposed Model) + XGBClassifier	0.718	0.739	0.672	0.763	0.704	0.718	1241 + 1.36
(MobileNetV2 + Proposed Model) + AdaBoostClassifier	0.747	0.758	0.727	0.767	0.742	0.747	841 + 0.34
(InceptionResNetV2 + Proposed Model) + AdaBoostClassifier	0.561	0.683	0.229	0.893	0.343	0.561	2740 + 0.17

Bold values indicate the highest values.

**Table 12:** Comparison between the proposed model and existing approaches in the literature.

Study	Model	Class	Accuracy	Precision	Sensitivity	Specificity	F1 Score	AUC
Goyal et al. [17]	Ensemble CNN with SVM classifier	Ischemia	0.903	0.918	0.886	0.921	0.902	0.904
		Infection	0.727	0.735	0.709	0.744	0.722	0.731
Al-Garaawi et al. [20]	GoogLNet CNN	Ischemia	0.92	0.94	0.93	0.90	0.93	0.97
		Infection	0.73	0.73	0.74	0.71	0.76	0.81
Proposed Work	(EfficientNetB0 + Head Model) + LogisticRegression	Ischaemia	0.967	0.967	0.968	0.967	0.967	0.967
	(EfficientNetB0 + Head Model) + AdaBoostClassifier	Infection	0.927	0.934	0.919	0.936	0.927	0.927

**Table 13:** Comparison of results each stage of ischemia classification.

Name of the Pre-Trained Model	Accuracy	Name of the Pre-Trained Model + Proposed Head Model	Accuracy	Name of the Pre-Trained Model + Proposed Head Model + ML	Accuracy
EfficientNetB0	0.947	EfficientNetB0 + Proposed Model	0.965	(EfficientNetB0 + Proposed Model) + LogisticRegression	**0.967**
ResNet101	0.925	ResNet101 + Proposed Model	0.933	(ResNet101 + Proposed Model) + XGBClassifier	**0.935**
DenseNet121	0.899	DenseNet121 + Proposed Model	0.902	(DenseNet121 + Proposed Model ) + KNeighborsClassifier	**0.911**
VGG16	**0.886**	VGG16 + Proposed Model	0.878	(VGG16 + Proposed Model) + KNeighborsClassifier	0.882
InceptionV3	**0.819**	InceptionV3 + Proposed Model	0.775	(InceptionV3 + Proposed Model) + XGBClassifier	0.777
MobileNetV2	**0.832**	MobileNetV2 + Proposed Model	0.785	(MobileNetV2 + Proposed Model) + AdaBoostClassifier	0.784
InceptionResNetV2	0.560	InceptionResNetV2 + Proposed Model	0.638	(InceptionResNetV2 + Proposed Model) + AdaBoostClassifier	**0.652**

Bold values indicate the highest values.

**Table 14:** Comparison of results of each stage of infection classification.

Name of the Pre-Trained Model	Accuracy	Name of the Pre-Trained Model + Proposed Head Model	Accuracy	Name of the Pre-Trained Model + Proposed Head Model + ML	Accuracy
EfficientNetB0	0.904	EfficientNetB0 + Proposed Model	0.919	(EfficientNetB0 + Proposed Model) + AdaBoostClassifier	**0.927**
ResNet101	0.896	ResNet101 + Proposed Model	**0.899**	(ResNet101 + Proposed Model) + LogisticRegression	0.900
DenseNet121	0.829	DenseNet121 + Proposed Model	0.868	(DenseNet121 + Proposed Model) + XGBClassifier	**0.874**
VGG16	0.827	VGG16 + Proposed Model	0.847	(VGG16 + Proposed Model) + LogisticRegression	**0.863**
InceptionV3	**0.763**	InceptionV3 + Proposed Model	0.707	(InceptionV3 + Proposed Model) + XGBClassifier	0.718
MobileNetV2	0.747	MobileNetV2 + Proposed Model	0.738	(MobileNetV2 + Proposed Model) + AdaBoostClassifier	0.747
InceptionResNetV2	0.535	InceptionResNetV2 + Proposed Model	**0.561**	(InceptionResNetV2 + Proposed Model) + AdaBoostClassifier	0.561

Bold values indicate the highest values.

## Data Availability

DFU-Part (B) dataset as utilized for the purpose of this research and it is available by the request http://www2.docm.mmu.ac.uk/STAFF/M.Yap/dataset.php (accessed on 16 July 2024). The source code available here: https://github.com/NoufAlmufadi/E-DFu-Net-with-ML/tree/main (accessed on 16 July 2024).

## References

[1] Jeffcoate W.J., Harding K.G. (2003). Diabetic foot ulcers. Lancet.

[2] Wild S., Roglic G., Green A., Sicree R., King H. (2004). Global prevalence of diabetes: Estimates for the year 2000 and projections for 2030. Diabetes Care.

[3] Noor S., Khan R.U., Ahmad J. (2017). Understanding diabetic foot infection and its management. Diabetes Metab. Syndr. Clin. Res. Rev..

[4] Armstrong D.G., Boulton A.J., Bus S.A. (2017). Diabetic foot ulcers and their recurrence. N. Engl. J. Med..

[5] Ananian C.E., Dhillon Y.S., Van Gils C.C., Lindsey D.C., Otto R.J., Dove C.R., Pierce J.T., Saunders M.C. (2018). A multicenter, randomized, single-blind trial comparing the efficacy of viable cryopreserved placental membrane to human fibroblast-derived dermal substitute for the treatment of chronic diabetic foot ulcers. Wound Repair Regen..

[6] Santilli J.D., Santilli S.M. (1999). Chronic critical limb ischemia: Diagnosis, treatment and prognosis. Am. Fam. Physician.

[7] Haque F., Reaz M.B.I., Chowdhury M.E.H., Ezeddin M., Kiranyaz S., Alhatou M., Ali S.H.M., Bakar A.A.A., Srivastava G. (2022). Machine learning-based diabetic neuropathy and previous foot ulceration patients detection using electromyography and ground reaction forces during gait. Sensors.

[8] Cao Z., Zeng Z., Xie J., Zhai H., Yin Y., Ma Y., Tian Y. (2023). Diabetic Plantar Foot Segmentation in Active Thermography Using a Two-Stage Adaptive Gamma Transform and a Deep Neural Network. Sensors.

[9] Albers M., Fratezi A.C., De Luccia N. (1992). Assessment of quality of life of patients with severe ischemia as a result of infrainguinal arterial occlusive disease. J. Vasc. Surg..

[10] Prompers L., Huijberts M., Apelqvist J., Jude E., Piaggesi A., Bakker K., Edmonds M., Holstein P., Jirkovska A., Mauricio D. (2007). High prevalence of ischaemia, infection and serious comorbidity in patients with diabetic foot disease in Europe. Baseline results from the Eurodiale study. Diabetologia.

[11] Lipsky B.A., Berendt A.R., Cornia P.B., Pile J.C., Peters E.J., Armstrong D.G., Deery H.G., Embil J.M., Joseph W.S., Karchmer A.W. (2012). 2012 Infectious Diseases Society of America clinical practice guideline for the diagnosis and treatment of diabetic foot infections. Clin. Infect. Dis..

[12] Lavery L.A., Armstrong D.G., Wunderlich R.P., Tredwell J., Boulton A.J. (2003). Diabetic foot syndrome: Evaluating the prevalence and incidence of foot pathology in Mexican Americans and non-Hispanic whites from a diabetes disease management cohort. Diabetes Care.

[13] Leightley D., McPhee J.S., Yap M.H. (2016). Automated analysis and quantification of human mobility using a depth sensor. IEEE J. Biomed. Health Inform..

[14] Goyal M., Reeves N.D., Rajbhandari S., Yap M.H. (2018). Robust methods for real-time diabetic foot ulcer detection and localization on mobile devices. IEEE J. Biomed. Health Inform..

[15] Cruz-Vega I., Hernandez-Contreras D., Peregrina-Barreto H., Rangel-Magdaleno J.d.J., Ramirez-Cortes J.M. (2020). Deep learning classification for diabetic foot thermograms. Sensors.

[16] Cassidy B., Reeves N.D., Pappachan J.M., Gillespie D., O’Shea C., Rajbhandari S., Maiya A.G., Frank E., Boulton A.J., Armstrong D.G. (2021). The DFUC 2020 dataset: Analysis towards diabetic foot ulcer detection. touchREVIEWS Endocrinol..

[17] Goyal M., Reeves N.D., Rajbhandari S., Ahmad N., Wang C., Yap M.H. (2020). Recognition of ischaemia and infection in diabetic foot ulcers: Dataset and techniques. Comput. Biol. Med..

[18] Amin J., Sharif M., Anjum M.A., Khan H.U., Malik M.S.A., Kadry S. (2020). An integrated design for classification and localization of diabetic foot ulcer based on CNN and YOLOv2-DFU models. IEEE Access.

[19] Al-Garaawi N., Ebsim R., Alharan A.F., Yap M.H. (2022). Diabetic foot ulcer classification using mapped binary patterns and convolutional neural networks. Comput. Biol. Med..

[20] Al-Garaawi N., Harbi Z., Morris T. (2022). Fusion of Hand-crafted and Deep Features for Automatic Diabetic Foot Ulcer Classification. TEM J..

[21] Xu Y., Han K., Zhou Y., Wu J., Xie X., Xiang W. (2021). Classification of Diabetic Foot Ulcers Using Class Knowledge Banks. Front. Bioeng. Biotechnol..

[22] Das S.K., Roy P., Mishra A.K. (2022). Recognition of ischaemia and infection in diabetic foot ulcer: A deep convolutional neural network based approach. Int. J. Imaging Syst. Technol..

[23] Shorten C., Khoshgoftaar T.M. (2019). A survey on image data augmentation for deep learning. J. Big Data.

[24] Hossin M., Sulaiman M.N. (2015). A review on evaluation metrics for data classification evaluations. Int. J. Data Min. Knowl. Manag. Process.

[25] Algeffari M., Alsharidah M. (2022). Rosuvastatin-Induced Oral Ulcer: A Case Report and Review of Literature. Case Rep. Dent..

[26] Abdellatif A.A., Rugaie O.A., Alhumaydhi F.A., Tolba N.S., Mousa A.M. (2023). Eco-Friendly Synthesis of Silver Nanoparticles by Nitrosalsola vermiculata to Promote Skin Wound Healing. Appl. Sci..

